# Improving the Age Estimation Efficiency by Calculation of the Area Ratio Index Using Semi-Automatic Segmentation of Knee MRI Images

**DOI:** 10.3390/biomedicines11072046

**Published:** 2023-07-20

**Authors:** Tatjana Matijaš, Ana Pinjuh, Krešimir Dolić, Darijo Radović, Tea Galić, Dunja Božić Štulić, Frane Mihanović

**Affiliations:** 1 University Department of Health Studies, University of Split, 21000 Split, Croatia; tmatijas@ozs.unist.hr (T.M.); kdolic@mefst.hr (K.D.); darijo.radovic@medikol.hr (D.R.); 2 Faculty of Mechanical Engineering, Computing and Electrical Engineering, University of Mostar, 88000 Mostar, Bosnia and Herzegovina; ana.pinjuh@fsre.sum.ba; 3 Department of Diagnostic and Interventional Radiology, University Hospital of Split, 21000 Split, Croatia; 4 School of Medicine, University of Split, 21000 Split, Croatia; 5 Polyclinic Medikol, 21000 Split, Croatia; 6 Department of Prosthodontics, Study of Dental Medicine, School of Medicine, University of Split, 21000 Split, Croatia; tea.galic@mefst.hr; 7 Faculty of Electrical Engineering, Mechanical Engineering and Naval Architecture (FESB), University of Split, 21000 Split, Croatia; dgotovac@fesb.hr

**Keywords:** magnetic resonance imaging, knee joint, ossifying epiphyses, age estimation, femur, segmentation, computer vision

## Abstract

The knee is an anatomical structure that can provide a great deal of data for research on age estimation. The aim of this study was to evaluate and apply a method for semi-automatic measurements of the area under the growth plate closure of the femur distal epiphysis and the growth plate closure itself on the 2D coronary slices using T2 weighted images (T2WI) generated on magnetic resonance (MRI) devices of different technical and technological characteristics. After the semi-automatic segmentation of the femur distal epiphysis under the growth plate closure and the growth plate closure itself, the areas of the measured closures were calculated using MATLAB version: 9.12. (R2022a), MathWorks Inc., Natick, MA, USA, for each individual coronal slice. The area ratio index (ARI) was calculated as the ratio between the area under the growth plate closure of the femur distal epiphysis and the growth plate closure itself. The study sample consisted of 27 female and 23 male Caucasian participants aged 10 to 26 years. A total of 339 T2WI images were used for ARI calculations. There was a positive correlation between chronological age and the average ARI measured by three independent observers (r = 0.8280, *p* < 0.001). Multiple regression analysis did not show any significant impact of the technical and technological characteristics of the MRI devices on ARI. The results of this study showed that ARI could serve as a useful tool for age estimation using knee MRI as well as for the further development of artificial intelligence (AI) applications.

## 1. Introduction

The Study Group on Forensic Age Diagnostics (AGFAD) created the guidelines and criteria for age estimation in living individuals, which include a consensus among scientists about the most appropriate methods to use in specific situations, drawing up recommendations for age estimation, and institutionalizing quality control with special attention to sensitive legal and ethical implications [1]. Forensic anthropology is a part of forensic science, addressing research areas related to populations and demographic characteristics such as age, sex, stature, and race for different purposes [2]. Clear information concerning the accuracy of age estimation using dental and skeletal methods should be available, and in cases of age estimation in living individuals, the principles of medical ethics and legal regulations must be considered. Some dental and skeletal features successfully determine the end of growth as proper specific markers for restricted age ranges, which go from adolescence to early adulthood, while in some cases age estimation assessment methods are recommended [2,3]. The potential risks of radiological examination must be avoided, with the recommendation that no unnecessary or overdosed X-rays be used in the age assessment [4,5,6]. It can only be performed as a part of a judicial order since the subject is exposed to ionizing radiation, which can increase the risk of long-term effects like cancer [7]. Recent studies have made significant breakthroughs in the application of noninvasive imaging procedures in estimating the age of living subjects, predominantly magnetic resonance imaging (MRI) and ultrasound examination [7,8,9,10,11,12,13,14,15,16,17].

The knee is an anatomical structure that can provide a great deal of data for research on age estimation. The knee joint is composed of the articulation surfaces of three different bones: the distal femoral epiphysis, the proximal tibial epiphysis, and the patella. Non-invasive studies evaluating the age assessment based on MRI recordings have shown that the ossification of growth plate closure in the knee correlates with the chronological age of young individuals [7,18,19,20,21]. 

Age estimation is, therefore, never an exact science [22]. Many authors have concluded that the combination of various methods is the correct way to reduce uncertainty and increase the overall reliability of age estimation [22,23].

The epiphyseal fusion and ossification processes in the knee can be arranged into multiple stages. The correlation between the assigned stage and the age of the examined subject can then be analyzed. Most available studies are based on qualitative comparisons. Grading knee skeletal maturation is based on the appearance of the epiphyseal line, which presents the site of growth in a long bone. The epiphyseal line is a fine structure consisting of mesenchymal cells in different maturation stages and becomes thinner and thinner throughout the process of skeletal maturation before it disappears and endochondral ossification interrupts [7,18,19]. Dedouit et al. defined a five-stage system for the proximal tibia using T2WI MRI images [7], and Jopp et al. [19] employed a three-stage system using T1WI MRI images instead. Results of the study from Dedouit et al. [7] showed that skeletal maturation of the distal femur and proximal tibia was correlated with age and earlier ossification in females than in males. In other studies, similar results were obtained. All grading systems classify the maturation into exclusive stages based on the characterization and delineation of the thin physeal line [18,20,21,24]. According to our knowledge and available information, the ratio of the surfaces of the growth plate closure and the bottom part of the femur under the growth plate closure has not been used in previous research. The choice of the knee region in this study was based on findings in the research with MRI images that reported the presence of cartilage signal intensity at the knee ossification centers in male and female individuals [20]. The recent findings in the bone age assessment research with MRI images of the knee also reported a uniform spatial pattern of maturation of ossification centers in the knee in both male and female individuals [25].

In a recent study, a quantitative approach has been increasingly used using computer-based methods to reduce the impact of observers on results, which speeds up the process and can create measurement standards [25].

Computer vision (CV), as a part of artificial intelligence (AI), is an interdisciplinary scientific field that focuses on the processing and analysis of visual data. It employs different techniques and algorithms for tasks like object detection, image segmentation, image classification, etc. The advantages of using computer vision have led to fully automated workflows and state-of-the-art results in the medical field [26,27]. A popular research area in CV is artificial neural networks (ANNs). Those are known to be feature selectors, meaning that they can learn to extract information relevant to a specific task [28,29].

In the field of radiology, different MRI devices are used, which are difficult to standardize, both in image data collection and image interpretation. The advantage of MRI technology is that it supports the manipulation of the image’s contrast, granting the possibility of highlighting different tissue types and allowing better visualization of ossification centers. Additionally, since MRI images are volumetric, more information can be extracted and analyzed when compared to 2D radiographs [25]. Different MRI devices and scanning protocols make it difficult to assess and score epiphyseal fissure fusion. The observer’s experience as well as the calibration of the device have an impact on image interpretation [30,31,32,33].

Therefore, the aim of this study was to evaluate and apply a method for semi-automatic measurements of the area under the growth plate closure of the femur distal epiphysis and the growth plate closure itself on the 2D coronary slices using T2WI MRI images from different MRI devices having different technical and technological characteristics and to see the potential influence of these characteristics on the measurement results. 

## 2. Materials and Methods

This was a cross-sectional, retrospective study based on the knee MRI images and the subjects’ chronological ages. A total of 339 T2WI MRI images of 50 Caucasian participants (female 27/54% and male 23/46%), aged 10 to 26 years (average age of female 15.852 and male 15.217 years), were analyzed (Table 1).

The images were collected from the PACS databases of two health institutions: the University Hospital of Split and Polyclinic Medikol in the region of Dalmatia, Croatia, between 2018 and 2022, with IRB approval from both institutions.

Exclusion criteria were fractures or dislocations involving the growth plate closure or those that showed surgical fixatives or implants near the diaphyseal-epiphyseal junction on the knee MRI images. Concerning the pathological history of participants (metabolic and endocrine diseases), as well as socioeconomic and sports activity status, data was not available in this retrospective study.

Different healthcare institutions use MRI devices with different technical and technological characteristics, which can lead to different results when applying computer segmentation and computer analysis for measuring areas on MRI images. In this study, we used T2WI MRI images. We used images from three different MRI devices: 1 T (Panorama, Philips, Best, The Netherlands), 9 subjects; 1.5 T (Avanto, Siemens, Erlangen, Germany), 30 subjects; and 3 T (Skyra, Siemens, Erlangen, Germany), 11 subjects.

The technical and technological characteristics of the MRI devices, as well as the imaging parameters used in the research, are presented in Table 2.

**Figure 1:**
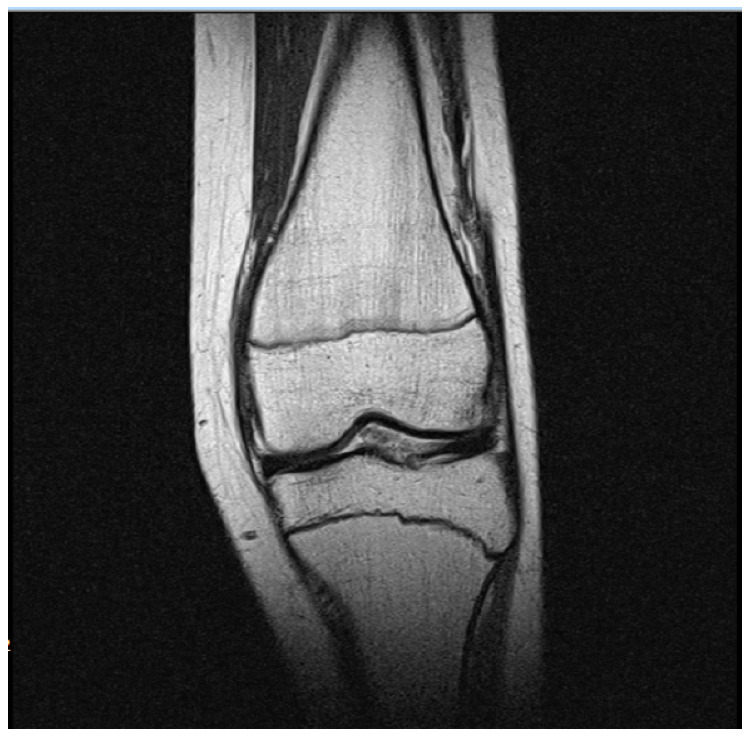
Original DICOM image of the patient’s knee from MRI T2WI.

Images were stored in DICOM (Digital Imaging and Communications in Medicine) format. DICOM is a standard for handling, storing, printing, and transmitting information in medical imaging [34]. From the MRI dataset of all patients, only T2WI were analyzed. Additionally, from all T2WI, we selected only those slices where growth plate closure as well as the femur were clearly visible (Figure 1), resulting in approximately seven slices per patient for analysis, determination, and segmentation of the growth plate closure.

For correct digital image segmentation, it is important to label each pixel in different regions that exhibit the same set of attributes [35]. Researchers use semi-automatic segmentation methods to perform knee MRI segmentation through human-computer interaction. Semi-automatic segmentation might be achieved using a number of algorithms [36]. The goal of image segmentation is to partition an image into homogeneous regions. We used image segmentation only from the lower part of the femur under the growth plate. For the purposes of this work, the following steps were executed:

- Segmenting the distal epiphysis of the femur under the growth plate closure.
- Segmenting the growth plate closure.

The first part was to manually segment the area of the femur, starting at the lower border of the growth plate closure and ending at the bottom of the femur. 

Three independent observers manually segmented the surface (area) of the distal epiphyseal fissure of the femur under the growth plate closure and the surface of the growth plate closure for each subject on all coronary slices where the epiphyseal fissure was clearly visible.

**Figure 2:**
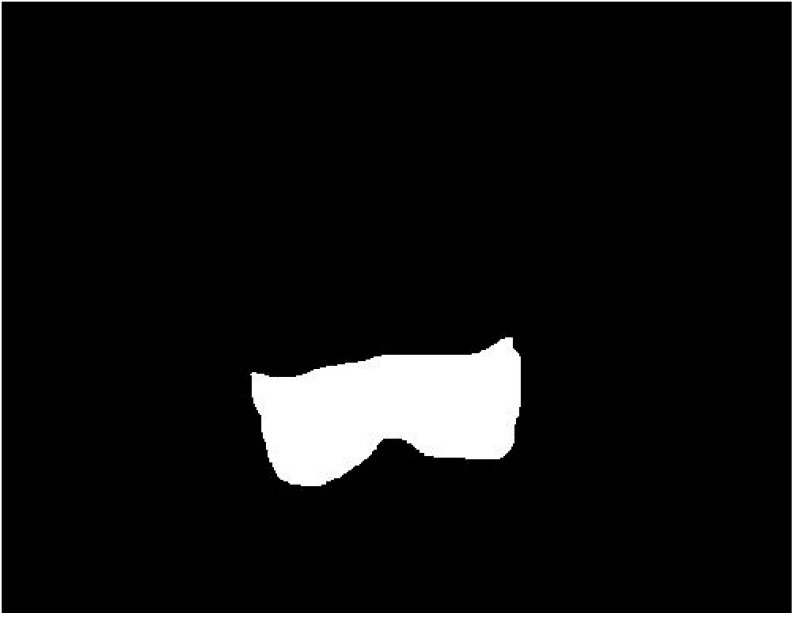
Segmented bottom part of the femur under the growth plate closure.

Hence, the result of the segmented region was entirely determined by the segmentation. For this step, the MATLAB version: 9.12. (R2022a), Natick, MA, USA, Image Segmenter app was used. The result of segmentation was a binary segmentation mask of this part of the femur (Figure 2). 

Next, the size of the mask needed to be calculated. For this task, technical meta-information about each image stored in the DICOM header was used, specifically the Pixel Spacing Attribute [37]. This attribute represented the physical distance in the patient between the centers of each pixel, specified by a numeric pair (row/column) in millimeters. This attribute showed the width and length of a single pixel and could be used to calculate pixel surface area. Finally, calculating the size of the segmented mask was a straightforward process once the number of pixels creating that mask and the value of their area were known.

The second step was semi-automatic segmentation of the growth plate closure area. First, the region of interest was determined by cropping the original image using the bounding box, which included the shown part of the epiphyseal crack and the part below the crack, which included the shown part of the growth plate closure and the part of the femur under the growth plate closure. All images were similar in patient orientation, and the region of interest was always in the center of the image, making it suitable for cropping using the same coordinates of the bounding box for all patients. 

Then, the thresholding technique was used to segment the growth plate closure after cropping the original image. Growth plate closure was visible as dark horizontal lines, whereas surrounding bone appeared bright; therefore, they were suitable for this method. The thresholding technique is a simple image processing technique widely used in image segmentation. It is based on the principle that all values within the boundary condition were preserved in the output image, and all values outside a set of boundary conditions were neglected [38]. 

Example of measuring/segmentation: Let I be the grayscale 2D image with pixel values in the range $\left[I_{min},\;I_{max}\right]$. Let $\left[T_{min},\;T_{max}\right]$ be the desired boundary condition range of pixel values to be preserved from the original image. The result of thresholding an original image is a binary output image $I_{output}$:

Ioutput=1, if Ix, y∈ Tmin, Tmax 0, otherwise

**Figure 3:**
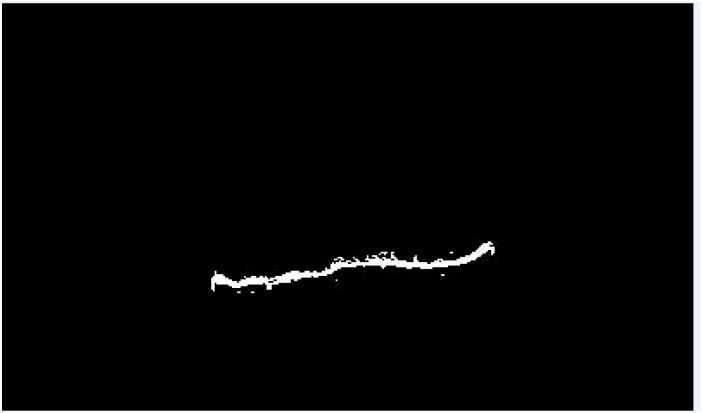
Segmented growth plate closure mask.

**Figure 4:**
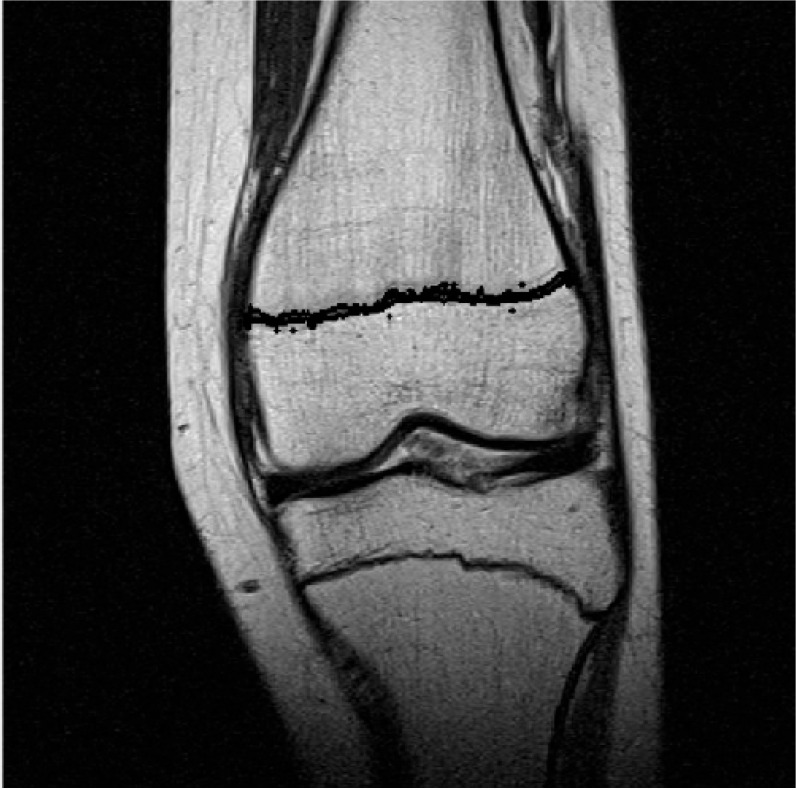
Outlined result of growth plate closure on the original image.

Additionally, some image filtering and morphological operations were used to enhance the resulting segmented binary mask (Figure 3) and to outline the result of growth plate closure on the original image (Figure 4). 

The area representing growth plate closure was calculated in the same manner as the area of the distal epiphysis of the femur using information from DICOM Tags that identifies the attribute, usually in the format (XXXX: XXXX) with hexadecimal numbers that can be further divided into DICOM Group Number and DICOM Element Number (Table 3).

After manual segmentation of the area of the femur under the growth plate closure (A_sf_) and area at the growth plate closure (A_se_) with computer correction for each observer, those areas were calculated by computer for each individual slice using the DICOM tag [0028:0030]. Pixel spacing for calculating the pixel area (Pix mm^2^) and the number of pixels (N Pix) in the binary segmentation mask area according to the formulas:

A_sf_ = Pix mm^2^ × N Pix

A_se_ = Pix mm^2^ × N Pix

For each slice (s) in the coronary plane where the growth plate closure was visible, the total area for the part of the femur under the growth plate closure (A_sf_) and the area of the growth plate closure (A_se_) were calculated. The total area of the bottom part of the femur under the growth plate closure (A_f_) as well as the total area of the growth plate closure (A_e_) were obtained by summing all the areas of individual slices according to the formulas:

$$A_{f}=\begin{array}{c} A_{\mathrm{sf}1}+A_{\mathrm{sf}2}+A_{\mathrm{sf}3\;\ldots } \end{array}$$

$$A_{e}=\begin{array}{c} A_{\mathrm{se}1}+A_{\mathrm{se}2}+A_{\mathrm{se}3\;\ldots } \end{array}$$

The area ratio index (ARI) was the result of the ratio of the areas of parts of the femur under the growth plate closure and growth plate closure according to the formula:

$$\mathrm{ARI}=\frac{A_{f}}{A_{e}}$$

To calculate the volume, DICOM tag [0018:0050] Slice Thickness (T_s_), the area of a part of the femur under the growth plate closure and the growth plate closure, as well as the total number of slices (N_s_) where the growth plate closure was visible according to the formulas, were used:

$$V_{f}=\begin{array}{c} A_{f}\times {\;T}_{s}\times {\;N}_{s} \end{array}$$

$$V_{e}=\begin{array}{c} A_{e}\times {\;T}_{s}\times {\;N}_{s} \end{array}$$

The volume ratio index (VRI) was the result of the ratio of the volume of the bottom part of the femur under the growth plate closure (V_f_) and the volume of the growth plate closure (V_e_) according to the formula:

$$\mathrm{VRI}=\;\frac{V_{f}\;}{V_{e}\;}$$

### Statistical Analysis and Data Management

MS Excel (Microsoft Office 365, Microsoft, Redmond, WA, USA) and MedCalc Statistical Software version 14.8.1 (MedCalc Software bvba, Ostend, Belgium) were used for all data management and statistical analyses. The significance level was set at *p* < 0.05.

Descriptive statistics were used to show the technical and technological characteristics of the MRI devices and imaging parameters used in the research.

Since the data was not normally distributed (Kolmogorov-Smirnov test), the Mann-Whitney *U* test was used to assess the differences between sex, age, and ARI. The Pearson’s correlation coefficient was used to relate the actual age with the average ARI. Inter-rater agreement (Kappa coefficient) was used to determine the reliability of measurements between three observers. Multiple regression analysis was used to assess the possible influence of the technical and technological characteristics of the MRI device on the ARI results.

## 3. Results

There was no statistically significant difference in age between females and males. Furthermore, there was no statistically significant difference in ARI results between females and males.

The results of the semi-automatic segmentation of the area of the growth plate closure and the lower part of the femur below the growth plate closure by three experienced observers (Obs1, Obs2, and Obs3) in the field of radiological imaging were computer processed according to the described method. Inter-rater agreements between three observers are presented in Table 4. 

The results obtained for the VRI were identical to the ARI values because the same number of layers where growth plate closure was visible was used for the volume calculation as well as for the area calculation with the same thickness of the slice. The results obtained from ARI were used for statistical processing.

The Pearson’s correlation coefficient was used to correlate the actual age (from the date of birth to the date of examination of the subject) with the average ARI of the three observers of the surface of the growth plate closure and the distal part of the femur under the growth plate closure. The results showed a highly positive correlation between age and the ARI results obtained by sex and in total for all subjects (Table 5).

The possible influence of age and technical and technological characteristics on ARI results was assessed by multiple regression analysis (Table 6). The regression model shows a statistically significant impact of age on ARI, while the technical and technological characteristics of MRI devices do not have a statistically significant impact on ARI. The coefficient of determination R^2^ was 0.712, the F-ratio was 12.669, and the statistical significance was *p* < 0.0001.

## 4. Discussion

The results of our study demonstrated that ARI, as a method of evaluating epiphyseal fissure fusion, showed a high correlation with the age of all subjects (r = 0.828) and both sexes: females (r = 0.797) and males (r = 0.904), respectively. The obtained results of the ratio index for ARI and VRI were identical, which proved that the method of calculating the area ratio of the growth plate closure and the bottom part of the femur under the growth plate closure of the femur can be used for 2D and 3D imaging modes on MRI. 

The results obtained from this study showed that there were no statistically significant differences in the use of different MRI devices with different technical-technological characteristics with the obtained area ratio index (ARI) result between the surface of the growth plate closure and the lower part of the femur under the growth plate closure, while the age of the subjects showed significant influence on ARI. 

Results from previous studies performed in different institutions on different MRI modalities with different readers and different image scan parameters are not directly comparable. MRI is the modality that is the most difficult to standardize, both in terms of image reading and image acquisition [31,32]. Objective assessment of MRI signals is difficult because readers tend to perceive the same image intensity differently depending on surrounding background intensities [33]. The reader’s experience and calibration of the reading structure also influence the interpretation of the images [26]. Factors that could influence the results when comparing MRI image analyses are the different strengths of the MRI magnetic field, pixel size, number of pixels on the image, image matrix, slice thickness, total slices of the study area, and total width of the study area. Saint-Martin et al. argued that the magnetic field had no effect, and there is no comparative study showing the effect of magnetic field differences on the data [11]. 

The European Commission (EC) suggested, based on the recommendations of the European Asylum Support Office (EASO), that the assessment should first apply radiation-free medical methods [39]. MRI as a non-invasive method in the analysis of ossification of growth plate closure for age assessment is recommended by AFGAD (1).

The semi-automatic segmentation methods on digital MRI images were used in previous studies to improve research efficiency [40,41]. In this study, we used the manual segmentation performed by observers, corrected with computer segmentation.

Deep learning has grown exponentially in the domain of computer vision and depends a lot on the availability of high-quality images with labeled datasets by professionals to train, test, and validate algorithms. The limited availability of such datasets is commonly the limiting factor in research and projects [42]. 

Compared to other CV applications, CV applications for medical imaging analysis seem to have an advantage since medical images are generated and archived using the DICOM standard. They are mostly ideally captured, lacking occlusion and miss-orientation problems and minimizing distortion, deformation, and mis-illumination problems that other images generally present [43]. 

The results of semi-automatic segmentation and calculation of the areas of the growth plate closure area and the lower part of the femur under the growth plate closure showed a high intraclass correlation coefficient (0.9980). 

The limitations of this study might be the small sample size, although a total of 339 images were analyzed. Additionally, further work will be directed toward the collection of more data, which may improve the precision of our method. Considering the retrospective design of the study, there is a lack of information about sports activities, socioeconomic status, and possible diseases, especially endocrine diseases. This method was built upon data from healthy youth and young adult subjects, and the effects of disorders that can affect growth were not investigated.

## 5. Conclusions

The results of this study showed that using MRI devices with different technical and technological characteristics for measurements of the area under the growth plate closure of the femur distal epiphysis and the growth plate closure itself did not influence the results of the ARI used for age estimation. Therefore, such methods may guide future studies, help researchers decide on a preferred approach for specific cases, and contribute to multifactorial age estimation better than based on a single anatomical site. 

In addition, ARI can be used in the further development of the application of AI in age estimation in order to develop precise and accurate methods for the application of computer vision algorithms.

## Figures and Tables

**Table 1:** Study population according to age and sex.

Sex	N	Mean ± SD	95% CI Mean	SEM	Median	Min	Max	95% CI Median
Female	27	15.852 ± 4.194	[14.193;17.511]	0.807	14.000	10.000	26.000	[13.953;17.000]
Male	23	15.217 ± 3.397	[13.748;16.686]	0.708	15.000	11.000	23.000	[13.000;16.651]
Total	50	15.560 ± 3.823	[14.473;16.647]	0.540	15.000	10.000	26.000	[14.000;16.000]

**Table 2:** The technical and technological characteristics of the MRI devices used and imaging parameters.

MRI Device	N	Magnetic Field Strength (T)	Pixel Size (mm)	Image Matrix (pix.)	Slice Thickness (mm)	
Skyra, Siemens	11	3 T	0.484–0.500	320 × 320	3–4	
Avanto, Siemens	30	1.5 T	0.273–0.446	512 × 512	3–4	
Panorama, Philips	9	1.0 T	0.484–0.625	288 × 288	3–4	
**Technological Characteristics** **of the MRI Devices**	**N**	**Mean ± SD**	**95% CI Mean**	**Median**	**Min**	**Max**
Magnetic field strength (T)	50	1.740 ± 0.702	[1.541;1.939]	1.500	1.000	3.000
Pixel size (mm^2^)	50	0.180 ± 0.091	[0.154;0.206]	0.148	0.075	0.391
Total number of pixels on the image	50	189,173.760 ± 82,265.344	[165,794.208;212,553.312]	262,144.000	65,536.000	262,144.000
Image matrix area (mm^2^)	50	27,133.132± 4887.933	[25,743.991;28,522.272]	25,681.986	19,537.300	40,076.800
Slice thickness (mm)	50	3.760 ± 0.431	[3.637;3.883]	4.000	3.000	4.000
Total slices of the study area	50	6.780 ± 0.9–75	[6.503;7.057]	7.000	5.000	9.000
Total width of the study area (mm)	50	25.260 ± 3.193	[24.353;26.167]	24.000	18.000	32.000

**Table 3:** DICOM tags and descriptions of tags used in the research.

Group, Element	Title
[0028:0010]	Rows
[0028:0011]	Columns
[0028:0030]	Pixel Spacing
[0018:0050]	Slice Thickness
[0018:0087]	Magnetic Field Strength

**Table 4:** Inter-rater agreement.

	Kappa	95% CI
Obs1 vs. Obs2	0.959	0.953 to 0.965
Obs1 vs. Obs3	0.939	0.9154 to 0.963
Obs2 vs. Obs3	0.916	0.891 to 0.941

**Table 5:** Correlation between age and the obtained ARI results by sex.

	N	r	95% CI for r	*p*
Female	27	0.798	0.599 to 0.904	*p* < 0.0001
Male	23	0.904	0.783 to 0.959	*p* < 0.0001
Total	50	0.828	0.723 to 0.903	*p* < 0.0001

**Table 6:** Multiple regression values for the dependent variable ARI.

Independent Variables	Coefficient	SE	r_partial_	t	*p*
Constant	−25.444				
Age	1.759	0.217	0.785	8.119	<0.0001
Sex	0.198	1.733	0.018	0.114	0.909
Magnetic field strength T	−2.654	1.889	−0.214	−1.405	0.167
Pixel_size_mm^2^	−44.962	45.785	−0.152	−0.982	0.332
Image_matrix_surface_mm^2^	0.0002029	0.0002822	0.112	0.719	0.476
Number of pixels on the image	−0.00006196	0.00005585	−0.171	−1.109	0.274
Slice thickness mm	9.767	15.398	0.099	0.634	0.529
Total slices of the study area	3.239	7.406	0.068	0.437	0.664
Total width of the study area	−0.985	2.004	−0.076	−0.491	0.626

## Data Availability

The data presented in this study are available on request from the corresponding author.
